# Supplementary material for: Automatic segmentation of the spinal cord nerve rootlets
Source: Imaging Neurosci (Camb). 2024 Jul 2;2:imag-2-00218. doi: 10.1162/imag_a_00218 (PMC12272210; doi:10.1162/imag_a_00218)
Supplement: Supplementary Table 1 [file imag_a_00218-supp1.pdf]

Level	Fold 0	Fold 1	Fold 2	Fold 3	Fold 4	Fold all
C2	0.75 ± 0.07	**0.77 ± 0.06**	0.74 ± 0.09	0.75 ± 0.09	0.75 ± 0.09	0.77 ± 0.08
C3	0.72 ± 0.07	0.73 ± 0.07	0.70 ± 0.08	0.73 ± 0.07	0.71 ± 0.08	**0.74 ± 0.07**
C4	0.65 ± 0.14	**0.65 ± 0.10**	0.64 ± 0.14	0.63 ± 0.15	0.66 ± 0.12	0.65 ± 0.19
C5	0.56 ± 0.31	**0.66 ± 0.12**	0.55 ± 0.3	0.53 ± 0.31	0.57 ± 0.28	0.57 ± 0.28
C6	0.67 ± 0.15	0.67 ± 0.17	0.63 ± 0.17	0.65 ± 0.16	**0.67 ± 0.14**	0.67 ± 0.15
C7	0.65 ± 0.12	0.59 ± 0.2	0.64 ± 0.14	0.64 ± 0.12	**0.66 ± 0.13**	**0.66 ± 0.13**
C8	0.60 ± 0.20	0.39 ± 0.33	**0.62 ± 0.20**	0.58 ± 0.20	0.59 ± 0.22	0.61 ± 0.19
**mean**	0.66 ± 0.15	0.64 ± 0.15	0.65 ± 0.16	0.65 ± 0.16	0.66 ± 0.15	**0.67 ± 0.16**
