# Supplementary material for: Automatic segmentation of the spinal cord nerve rootlets
Source: Imaging Neurosci (Camb). 2024 Jul 2;2:imag-2-00218. doi: 10.1162/imag_a_00218 (PMC12272210; doi:10.1162/imag_a_00218)
Supplement: Supplementary Table 2 [file imag_a_00218-supp2.pdf]

Level	**sub-barcelona01**					**sub-brnoUhb03**					**sub-amu02**					**sub-007**					**sub-010**					**COV**
	rater1	rater2	rater3	rater4	COV	rater1	rater2	rater3	rater4	COV	rater1	rater2	rater3	rater4	COV	rater1	rater2	rater3	rater4	COV	rater1	rater2	rater3	rater4	COV	**mean**
C2	43.8	45.0	44.2	45.0	1.4	36.7	37.4	36.7	36.9	1.0	44.3	43.9	43.9	43.9	0.5	46.7	48.2	46.4	47.9	1.9	46.5	45.6	44.4	46.5	2.2	1.38
C3	58.8	59.6	59.2	60.1	0.9	47.9	50.2	50.2	49.9	2.2	55.6	55.6	56.9	56.0	1.1	63.5	64.7	65.3	63.2	1.6	60.3	61.5	60.3	61.5	1.2	1.38
C4	76.6	76.6	77.0	77.8	0.7	62.7	64.7	64.7	63.4	1.5	71.7	72.5	71.7	71.7	0.6	78.8	81.9	80.6	77.9	2.2	76.7	79.1	76.4	79.8	2.2	1.45
C5	93.9	95.1	94.7	94.3	0.5	77.5	78.7	77.5	79.0	1.0	86.0	87.7	87.2	88.1	1.0	97.1	100.1	98.6	98.0	1.3	94.6	95.9	95.6	95.0	0.6	0.89
C6	109.2	110.0	109.2	109.6	0.4	90.5	91.8	91.0	91.0	0.6	100.1	100.1	99.3	100.5	0.5	115.1	115.1	114.4	115.1	0.3	110.3	112.8	112.8	112.1	1.0	0.55
C7	123.4	123.4	122.6	123.0	0.3	103.0	105.7	104.6	104.9	1.1	111.9	112.4	112.8	113.2	0.5	129.4	129.7	133.4	129.7	1.5	127.7	128.6	129.0	129.0	0.5	0.76
C8	138.6	140.3	139.5	139.9	0.5	118.3	117.5	118.7	119.3	0.6	126.7	128.1	127.7	127.7	0.5	149.4	150.0	150.0	148.8	0.4	142.5	144.3	143.7	145.3	0.8	0.57
